# Real-world effectiveness of Avelumab maintenance in advanced urothelial carcinoma: results from the Italian multicenter MALVA study (Meet-URO 25)

**DOI:** 10.1093/oncolo/oyaf388

**Published:** 2025-11-20

**Authors:** Giandomenico Roviello, Elisabetta Gambale, Irene De Gennaro Aquino, Marco Maruzzo, Carlo Messina, Ismaela Anna Vascotto, Virginia Rossi, Davide Bimbatti, Elisa Erbetta, Marco Messina, Alessia Mennitto, Sara Elena Rebuzzi, Cecilia Nasso, Chiara Mercinelli, Brigida Anna Maiorano, Martina Fanelli, Mariella Sorarù, Federico Scolari, Marinella Micol Mela, Luca Galli, Alessia Salfi, Mimma Rizzo, Silvia Puglisi, Valentina Orlando, Giuseppe Fornarini, Alessandro Rametta, Patrizia Giannatempo, Linda Cerbone, Laura Doni, Serena Pillozzi, Lorenzo Antonuzzo

**Affiliations:** Department of Health Sciences, Section of Clinical Pharmacology and Oncology, University of Florence, Florence 50139, Italy; Department of Experimental and Clinical Medicine, University of Florence, Florence 50139, Italy; Clinical Oncology, Careggi University Hospital, Florence 50139, Italy; Department of Experimental and Clinical Medicine, University of Florence, Florence 50139, Italy; Clinical Oncology, Careggi University Hospital, Florence 50139, Italy; Istituto Oncologico Veneto, IOV—IRCCS, Padova 50139, Italy; Clinical Oncology, Ospedale Arnas Civico, Palermo, Italy; Department of Experimental and Clinical Medicine, University of Florence, Florence 50139, Italy; Clinical Oncology, Careggi University Hospital, Florence 50139, Italy; Clinical Oncology, Careggi University Hospital, Florence 50139, Italy; Istituto Oncologico Veneto, IOV—IRCCS, Padova 50139, Italy; Istituto Oncologico Veneto, IOV—IRCCS, Padova 50139, Italy; Department of Surgery, Oncology and Gastroenterology, University of Padua, Padua, Italy; Clinical Oncology, Ospedale Arnas Civico, Palermo, Italy; Division of Oncology, University Hospital Maggiore della Carità, Novara, Italy; Medical Oncology Unit 2, Ospedale Molinette Azienda Ospedaliero-Universitaria Città della Salute e della Scienza di Torino Corso Bramante 88, 10126, Torino, Italy; Medical Oncology, Ospedale Santa Corona, 17027 Pietra Ligure, Italy; Medical Oncology Department, IRCCS San Raffaele Hospital, Milan, Italy; Vita-Salute San Raffaele University, Milan, Italy; Medical Oncology Department, IRCCS San Raffaele Hospital, Milan, Italy; Department of Oncology, University Hospital of Udine, Udine, Italy; Ospedale di Camposampiero, U.O. Oncologia, Camposampiero, Italy; Department of Biomedical, Experimental and Clinical Sciences, University of Florence, Florence, Italy; Clinical Oncology, Careggi University Hospital, Florence 50139, Italy; Medical Oncology Unit 2, Azienda Ospedaliero-Universitaria Pisana, Pisa, Italy; Medical Oncology Unit 2, Azienda Ospedaliero-Universitaria Pisana, Pisa, Italy; Oncologia Medica Universitaria Azienda Ospedaliera Universitaria Consorziale, Policlinico di Bari piazza Giulio Cesare, 11, 70124, Bari, Italy; IRCCS Ospedale Policlinico San Martino, Genoa, Italy; Department of Oncology, Ospedale Maggiore, Trieste, Italy; IRCCS Ospedale Policlinico San Martino, Genoa, Italy; Genitourinary Medical Oncology Department, Fondazione IRCCS Istituto Nazionale dei Tumori, Milan, Italy; Genitourinary Medical Oncology Department, Fondazione IRCCS Istituto Nazionale dei Tumori, Milan, Italy; Department of Medical Oncology, San Camillo Forlanini Hospital, Rome, Italy; Clinical Oncology, Careggi University Hospital, Florence 50139, Italy; Department of Experimental and Clinical Medicine, University of Florence, Florence 50139, Italy; Clinical Oncology, Careggi University Hospital, Florence 50139, Italy; Department of Experimental and Clinical Medicine, University of Florence, Florence 50139, Italy; Clinical Oncology, Careggi University Hospital, Florence 50139, Italy

**Keywords:** Avelumab, immunotherapy, prognostic score, urothelial cancer

## Abstract

**Importance:**

Avelumab maintenance therapy improves survival in patients with advanced urothelial carcinoma who respond to first-line platinum-based chemotherapy. However, real-world evidence on its effectiveness and on the prognostic value of baseline clinical factors remains limited.

**Objective:**

To evaluate the real-world effectiveness of Avelumab maintenance therapy and to identify baseline prognostic factors associated with clinical outcomes.

**Design:**

Multicenter prospective observational study including a retrospective cohort of patients treated before study initiation.

**Setting:**

Several Italian oncology centers participating between 2021 and 2023.

**Participants:**

A total of 251 patients with advanced or metastatic urothelial carcinoma who received Avelumab maintenance therapy after achieving disease control with first-line platinum-based chemotherapy.

**Intervention(s) or Exposure(s):**

Avelumab maintenance therapy administered according to clinical practice. Baseline clinical variables, including ECOG performance status, metastatic sites, and corticosteroid use, were assessed for prognostic significance.

**Main Outcome(s) and Measure(s):**

Primary outcomes were overall survival (OS) and progression-free survival (PFS). Secondary outcomes included overall response rate (ORR) and disease control rate (DCR). Prognostic factors were evaluated using multivariate Cox regression.

**Results:**

Median OS was 22.4 months, and median PFS was 7 months. The ORR and DCR were 26.75% and 69.30%, respectively. Independent predictors of worse OS were ECOG ≥1 (HR 1.57), bone metastases (HR 1.88), brain metastases (HR 7.02), and baseline corticosteroid use (HR 2.42). An exploratory prognostic score integrating ECOG status, bone metastases, and steroid use stratified patients into three groups with significantly different outcomes: median OS was 39.9 months (no adverse factors), 14.1 months (one factor), and 8.4 months (two or more factors), with corresponding ORRs of 33.6%, 28.0%, and 6.9%.

**Conclusions and Relevance:**

In this large real-world cohort, Avelumab maintenance confirmed its effectiveness and safety in advanced urothelial carcinoma. A simple exploratory prognostic score based on ECOG performance status, bone metastases, and baseline corticosteroid use successfully stratified patients by survival outcomes and may support personalized treatment strategies in clinical practice.

Implications for practice Avelumab maintenance confirmed its effectiveness and safety in a real-world Italian cohort of patients with advanced urothelial carcinoma. Our study highlights the impact of simple clinical variables (ECOG status, bone metastases, corticosteroid use) on survival, which may help clinicians in patient stratification and treatment decision-making. These findings reinforce the role of avelumab maintenance as the standard of care after platinum-based chemotherapy while emphasizing the need for prospective validation of prognostic markers.

## Introduction

Urothelial carcinoma is the most common malignancy of the urinary tract and, in its advanced or metastatic stage, is associated with a poor prognosis despite recent therapeutic advances.^1^ Historically, platinum-based chemotherapy has represented the standard first-line treatment; however, its efficacy remains limited in terms of both duration of response and long-term survival.^2^

The advent of immunotherapy, particularly immune checkpoint inhibitors such as the programmed death-ligand 1 (PD-L1) Avelumab, has substantially changed the therapeutic approach to this disease. The pivotal JAVELIN Bladder 100 trial was the first to demonstrate that Avelumab, administered as maintenance therapy in patients without progression following platinum-based chemotherapy, significantly improves overall survival compared to best supportive care alone.^3^ These findings led to the approval of Avelumab as the standard of care in this setting.

Despite these promising results, applying maintenance therapy in everyday clinical practice presents new challenges. Patients treated outside clinical trials often exhibit more heterogeneous characteristics, including relevant comorbidities, compromised performance status, and variability in treatment management.^4^ Moreover, limited real-world data are available regarding the prognostic impact of clinical variables such as Eastern Cooperative Oncology Group (ECOG) performance status ≥1, the presence of bone or brain metastases, and concomitant corticosteroid use—all of which may adversely affect avelumab efficacy.

This Italian multicenter study Maintenance with AVeLumAb ([MALVA] in advanced urothelial neoplasms in response to first-line chemotherapy: a multicenter prospective observational study including a retrospective cohort of patients treated before study initiation (Meet-URO 25))^5^ aimed to assess the real-world effectiveness and safety of Avelumab as maintenance therapy in patients with advanced or metastatic urothelial carcinoma. Particular focus was placed on identifying clinical factors associated with unfavorable prognosis and evaluating their influence on survival outcomes.

## Patients and methods

This was a multicenter prospective observational study including a cohort of patients treated before study initiation. This study included patients with advanced urothelial carcinoma who received Avelumab as maintenance therapy following a response to platinum-based chemotherapy across several centers in Italy. Inclusion criteria were Age > 18 years; histological or cytological evidence of urothelial carcinoma; radiological evidence of locally advanced or metastatic urothelial carcinoma with no disease progression (after 4-6 cycles of first-line platinum-based chemotherapy) and signed informed consent. Exclusion criteria were: disease progression during or following first-line chemotherapy and prior treatment with immune checkpoint inhibitors. Eligible patients initiated Avelumab between 2021 and 2023. The data cutoff date was April 15, 2025.

Patient data—demographics, tumor characteristics, and treatment history—were collected through an institutional review board-approved review of electronic medical records. Demographic variables included age, sex, and race; treatment-related information comprised prior cystectomy, previous systemic therapies, baseline laboratory values, and response assessments. Corticosteroid use at baseline was recorded regardless of indication (eg, brain metastases, bone pain, or other cancer- and comorbidity-related symptoms such as fatigue, anorexia, or COPD).

Clinical outcomes included disease control rate (DCR; defined as the proportion of patients achieving complete response [CR], partial response [PR], or stable disease [SD] per RECIST v1.1^6^), overall response rate (ORR; percentage of CR or PR), progression-free survival (PFS; from Avelumab initiation to radiographic/clinical progression or death), and overall survival (OS; from treatment start to death from any cause). Patients without progression or death by the last follow-up were censored. Responders were defined as those achieving CR, PR, or SD; non-responders were classified as those with progressive disease (PD).

Primary endpoints were OS and PFS; secondary endpoints included ORR and DCR. Statistical analyses were conducted using Stata 18.0 SE—Standard Edition. Overall survival (OS) was defined as the time from initiation of avelumab maintenance to death from any cause. Progression-free survival (PFS) was defined as the time from avelumab initiation to radiographic or clinical progression, or death. Median follow-up was estimated using the reverse Kaplan–Meier method. Categorical variables were summarized using ­frequencies and percentages; continuous variables were ­presented as medians and ranges. Dichotomization of continuous variables was performed using median values when appropriate. Chi-square tests were used for categorical associations. Kaplan–Meier estimates were used for survival analysis and compared via log-rank test. Multivariate Cox proportional hazards models were applied to explore independent prognostic factors.

In order to identify clinical predictors of efficacy of avelumab, patients were stratified into three exploratory prognostic stratification groups based on the presence of the following negative factors: bone metastases, ECOG performance status ≥1, and corticosteroid use prior to Avelumab initiation. Group 0 included patients without any of these factors; Group 1 included those with one factor; and Group 2-3 included those with two or more factors.

## Results

### Patient characteristics

A total of 251 patients with advanced or metastatic urothelial carcinoma were included (Table 1), among these, 62 (24.7%) were enrolled retrospectively and 189 (75.3%) prospectively, reflecting the retro-prospective design of the analysis. The median age was 72 years, with 38.2% of patients aged ≥75 years. Most patients were male (82.07%) and had ECOG performance status 0 (60.56%), and only 10 patients (3.9%) had ECOG ≥2, confirming that the vast majority of our cohort had preserved performance status at baseline. The primary tumor site was the bladder in most cases (*n* = 186), while 65 patients had upper urinary tract urothelial carcinoma. Histologically, 91.63% had pure urothelial carcinoma, with 8.37% showing urothelial carcinoma with histologic subtypes.

**Table 1.** Patient baseline characteristics.

Variables	Total 251 patients
**Age, median (range), years**	72 (38-88)
** ≥75 years; *n* (%)**	96 (38.2)
**Sex, *n* (%)**	
** Male**	206 (82.07)
** Female**	45 (17.93)
**ECOG performance status, *n* (%)**	
** 0**	152 (60.56)
** 1**	89 (35.46)
** ≥2**	10 (3.98)
**Primary tumor site, *n* (%)**	
** Bladder**	186 (74.10)
** Upper urinary tract**	65 (25.90)
**Hystology**	
** Urothelial**	230 (91.63)
** urothelial carcinoma with histologic subtypes**	21 (8.37)
**Site of metastasis, *n* (%)**	
** Lung**	78 (31.08)
** Bone**	66 (26.29)
** Liver**	35 (13.44)
** Lymph node**	192 (76.49)
** Brain**	3 (1.23)
** Other**	45 (18.4)
**Metastatic at diagnosis *n* (%)**	
** Yes**	108 (43.03)
** No**	143 (56.97)
**Comorbidities, *n* (%)**	
** Cardiovascular**	164 (65.34)
** Respiratory**	21 (8.37)
** Genitourinary**	23 (9.16)
** Diabetes**	44 (17.53)
** Dyslipidaemia**	64 (25.50)
** Autoimmune disease**	14 (5.58)
** ≥ 3 comorbidities**	30 (11.95)
**Prior treatment, *n* (%)**	
** Surgery**	109 (43.43)
** Radio chemotherapy**	2 (0.79)
**Prior chemotherapy regimen, *n* (%)**	
** Gemcitabine + cisplatin**	117 (46.80)
** Gemcitabine + carboplatin**	133 (53.20)
**Number of cycles of prior chemotherapy, *n* (%)**	
** 4**	179 (71.73)
** 5**	23 (9.16)
** 6**	49 (19.52)
**Best overall response to prior chemotherapy, *n* (%)**	
** CR**	12 (4.78)
** PR**	134 (53.39)
** SD**	105 (41.83)

The most frequent metastatic sites were lymph nodes (76.49%), lungs (31.08%), and bones (26.29%). At diagnosis, 43.03% of patients had metastatic disease. Common comorbidities included cardiovascular disease (65.34%), dyslipidemia (25.50%), and diabetes (17.53%); 11.95% of patients had three or more comorbidities.

Overall, 43.43% had undergone surgery. Prior chemotherapy consisted of gemcitabine plus cisplatin (46.80%) or carboplatin (53.20%), with a median of four cycles. The ORR to chemotherapy was 53.39%, and the SD rate was 41.83%.

### Response to Avelumab maintenance

**Figure 1.**
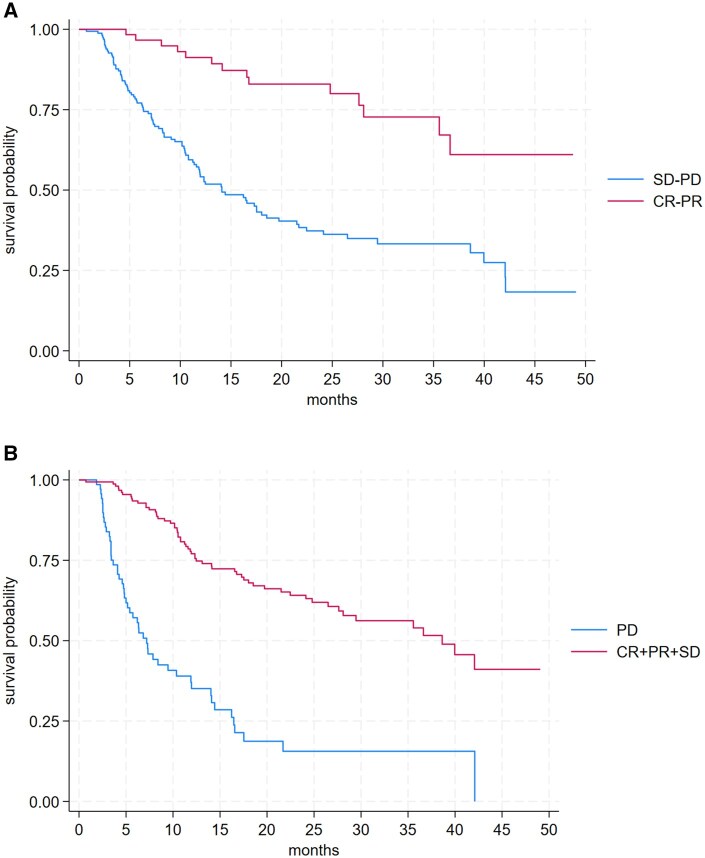
(A) Impact of disease control rate on overall survival. (B) Impact of objective response rate on overall survival.

After a median follow-up of 16 months, the ORR during Avelumab maintenance was 26.75% (CR 9.96%, PR 14.34%), while the DCR reached 69.30%. Median PFS was 7 months (95% CI: 5.6-9.3), and median OS was 22.4 months (95% CI: 16.5-36.6). Patients who achieved a CR or PR showed a median OS not reached (95% CI: 35.5–NR), compared to 14.1 months in non-responders (SD+PD). Including SD, median OS was 38.6 months in responders (CR+PR+SD) versus 7.2 months in patients with PD (Supplemental Table S1, Figure 1).

### Univariate and multivariate analysis for OS

**Figure 2.**
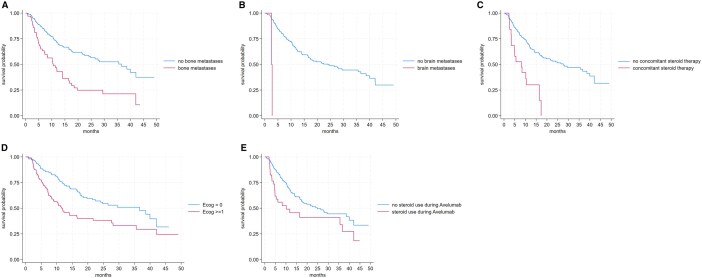
(A) Impact of bone metastases on overall survival. (B) Impact of brain metastases on overall survival. (C) Effect of concomitant steroid use on overall survival. (D) Prognostic impact of ECOG performance status on overall survival. (E) Effect of steroid use during Avelumab treatment on overall survival.

In the univariate analysis, factors significantly associated with shorter OS included ECOG ≥1 (HR 1.85; *P* = .001), bone metastases (HR 2.43; *P* < .001), brain metastases (HR 15.45; *P* < .001), concomitant steroid use (HR 3.32; *P* < .001), and steroid use during Avelumab (HR 1.75; *P* = .024) (Figure 2). Other data explored in the univariate analysis were reported in Table 2 and Supplemental Figure S1.

**Table 2.** Univariate for OS.

	HR	IC 95%	*P*
**≥75 years (yes vs no)**	0.82	0.56-1.19	.301
**Ecog (>=1 vs 0)**	**1.85**	**1.28-2.67**	**.001**
**Sex (male vs female)**	0.93	0.57-1.53	.789
**Cardiovascular comorbidity (yes vs no)**	0.92	0.63-1.35	.689
**Respiratory comorbidity (yes vs no)**	0.79	0.38-1.63	.537
**Genitourinary comorbidity (yes vs no)**	1.11	0.59-2.08	.736
**Diabetes (yes vs no)**	1.19	0.76-1.86	.429
**Dyslipidaemia (yes vs no)**	1.10	0.71-1.68	.663
**≥ 3 comorbidities (yes vs no)**	1.35	0.79-2.29	.266
**Autoimmune disorder (yes vs no)**	0.48	0.15-1.52	.216
**Previous BCG (yes vs no)**	1.14	0.73-1.78	.557
**Previous neoadjuvant cht (yes vs no)**	1.74	0.77-4.01	.179
**Previous surgery (yes vs no)**	1.18	0.82-1.69	.368
**Site of primary tumor (lower vs upper)**	0.63	0.32-1.26	.200
**Histology (urothelial vs urothelial carcinoma with histologic subtypes)**	0.94	0.49-1.80	.862
**Metastatic at diagnosis (yes vs no)**	1.02	0.70-1.49	.878
**Chemotherapy (cis+gem vs Carbo+gem)**	1.09	0.76-1.58	.611
**Cycles I line chemotherapy (>4 vs 4)**	1.02	0.68-1.51	.916
**Response I line chemotherapy (RC-PR vs SD)**	0.79	0.55-1.13	.208
**Lung metastasis (yes vs no)**	1.14	0.78-1.67	.480
**Lymph node metastasis (yes vs no)**	0.82	0.54-1.23	.347
**Bone metastasis (yes vs no)**	**2.43**	**1.67-3.55**	**<.001**
**Liver metastasis (yes vs no)**	1.58	0.97-2.57	.062
**Brain metastasis (yes vs no)**	**15.45**	**3.51-68.01**	**<.001**
**Other metastasis (yes vs no)**	1.20	0.76-1.89	.419
**Concomitant steroid therapy (yes vs no)**	**3.32**	**1.90-5.79**	**<.001**
**Dosage > 10 mg prednisone (yes vs no)**	0.35	0.10-1.18	.092
**Steroid use during Avelumab (yes vs no)**	**1.75**	**1.07-2.73**	**.024**
**Dosage > 10 mg prednisone (yes vs no)**	0.89	0.37-2.16	.813
**Concomitant metformin (yes vs no)**	1.07	0.64-1.79	.787
**Concomitant PPI (yes vs no)**	1.43	0.89-2.30	.137
**Concomitant antibiotics (yes vs no)**	1.12	0.52-2.41	.765
**Concomitant COXI (yes vs no)**	0.28	0.03-2.02	.208
**Radiotherapy during avelumab (yes vs no)**	0.96	0.59-1.58	.894
**Avelumab beyond progression (yes vs no)**	1.36	0.77-2.39	.277
**Tox precoce from avelumab (yes vs no)**	1.51	0.78-2.89	.212
**Hospitalization from avelumab tox (yes v no)**	2.17	0.87-5.36	.093
**Intestinal Tox (yes vs no)**	1.84	0.90-3.79	.094
**Hepatic Tox (yes vs no)**	2.01	0.49-8.20	.327
**Skin tox (yes vs no)**	0.93	0.40-2.12	.867
**Thyroid tox (yes vs no)**	0.60	0.29-1.24	.176
**Infusion reaction (yes vs no)**	1.88	0.69-5.12	.216
**Delayed immune-related adverse events (yes vs no)**	1.26	0.40-3.99	.686
**Calcium values (upper the median vs lower)**	0.79	0.53-1.17	.249
**Glycaemic values (upper the median vs lower)**	0.89	0.60-1.31	.565
**Creatinine values (upper the median vs lower)**	1.10	0.76-1.59	.587
**Albumin values (upper the median vs lower)**	0.80	0.50-1.28	.363
**LDH values (upper the median vs lower)**	1.01	0.64-1.59	.959
**PCR values (upper the median vs lower)**	0.72	0.38-1.39	.340

In the multivariate model, ECOG ≥1 (HR 1.57; *P* = .019), bone metastases (HR 1.88; *P* = .002), brain metastases (HR 7.02; *P* = .012), and concomitant steroid use (HR 2.42; *P* = .004) remained independently associated with worse OS (Table 3).

**Table 3.** Multivariate for OS.

	HR	IC 95%	*P*
**Ecog (>=1 vs 0)**	**1.57**	**1.07-2.30**	**.019**
**Bone metastasis (yes vs no)**	**1.88**	**1.25-2.83**	**.002**
**Brain metastasis (yes vs no)**	**7.02**	**1.54-31.88**	**.012**
**Concomitant steroid therapy (yes vs no)**	**2.42**	**1.33-4.40**	**.004**
**Steroid use during Avelumab (yes vs no)**	1.28	0.78-2.10	.311

Bold values is statistically significant.

### Treatment and toxicity

Avelumab was discontinued in 58.96% of patients, primarily due to disease progression (80.75%) or treatment-related toxicity (16.15%). Three treatment-related deaths were reported. Overall, 37.05% received a subsequent line of therapy, most commonly enfortumab vedotin, paclitaxel, or platinum-based combinations (Supplemental Table S2).

The most frequent adverse events included thyroid toxicity (9.16%), cutaneous (5.58%), gastrointestinal (4.38%), and infusion-related reactions (3.19%). Hospitalization was required in 3.59% of patients. Four cases (1.59%) of delayed immune-related adverse events were reported (Table 4). Baseline corticosteroid use was documented in 19/251 patients (7.6%): prednisone (*n* = 11), dexamethasone (*n* = 6), methylprednisolone (*n* = 1), betamethasone (*n* = 1). During avelumab therapy, 34/251 patients (13.6%) received steroids (prednisone *n* = 28, dexamethasone *n* = 3, methylprednisolone *n* = 3). Steroid therapy was not confined to patients with brain (*n* = 3; 1.2%) or bone metastases (*n* = 66; 26.3%), but was also prescribed for other cancer-related or comorbidity-related indications, indicating heterogeneous indications across the cohort (Supplemental Table S3; Table 1).

**Table 4.** Most relevant toxicity from avelumab.

Variables	Total 251 patients	Most relevant toxicities and grade
**Early toxicity from avelumab**	16 (6.37)	Infusion reaction; (4 pts)
		Myositis grade 3; (3 pts)
		Hepatitis grade 3; (2 pts)
		Autoimmune cholangitis G3; (1pts)
**Hospitalization from avelumab toxicity**	9 (3.59)	Myositis grade 3; (1 pt)
**Intestinal toxicity**	11 (4.38)	Diarrhea G2; (2 pts)
**Hepatic toxicity**	3 (1.20)	Hepatitis grade 3; (2 pts)
		Autoimmune cholangitis G3; (1pts)
**Skin toxicity**	14 (5.58)	Erythema G2; (1 pt)
		Pruritus G2(1 pt)
		Papular rash G2; (1 pt)
**Lung Toxicity**	2 (0.80)	Pneumonitis G3; (2 pys)
**Pancreatic toxicity**	3 (1.20)	Increase in pancreatic markers G2 (1 pt)
**Neurologic Toxicity**	2 (0.80)	Miastenia gravis G3; (1 pt)
**Pituitary toxicity**	2 (0.80)	Hypophysitis G2; (1 pt)
**Thyroid Toxicity**	23 (9,16)	Hypothyroidism G2; (10 pts)
**Other toxicity**	33 (13.15)	Infusion reaction; (8 pts)
		Myositis grade 3; (3 pts)
**Delayed immune-related adverse events**	4 (1.59)	NR
**Deaths probably related to avelumab**	3 (1.20)	NR

### Exploratory prognostic stratification subgroup analysis

Group 0 (no adverse prognostic factors) included 119 patients (47.4%), Group 1 (one factor) included 86 patients (34.3%), and Group 2-3 (two or three factors) included 46 patients (18.3%). The differences in patient’s characteristics according to exploratory prognostic stratification group were reported in Supplemental Table S4.

**Figure 3.**
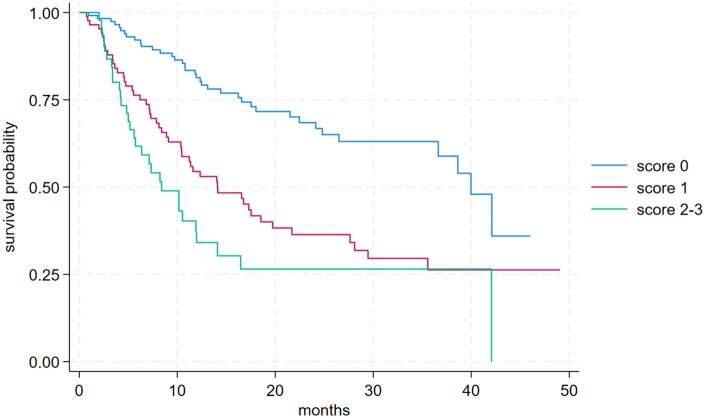
Overall survival according prognostic by score group.

ORR was highest in Group 0 (33.64%), followed by Group 1 (28.01%) and Group 2-3 (6.98%). DCR followed a similar trend: 81.31% in Group 0, 67.95% in Group 1, and 41.86% in Group 2-3. PD occurred in 54.35% of Group 2-3 patients, compared to 29.07% in Group 1 and 16.81% in Group 0 (Supplemental Figure S2). Median PFS was 16.9 months (95% CI: 8.4-27.1) in Group 0, 5.6 months (95% CI: 3.5-9.3) in Group 1, and 3.2 months (95% CI: 2.7-3.7) in Group 2-3 (Supplemental Figure S3). Median OS was 39.9 months (95% CI: 36.6–NR) in Group 0, 14.1 months (95% CI: 10.3-19.7) in Group 1, and 8.4 months (95% CI: 5.6-11.9) in Group 2-3 (Supplemental Table S5; Figure 3).

## Discussion

This retrospective multicenter study confirms the effectiveness of Avelumab maintenance therapy in a real-world cohort of patients with advanced or metastatic urothelial carcinoma. The observed median overall survival (OS) of 22.4 months and progression-free survival (PFS) of 7 months are consistent with the results of the JAVELIN Bladder 100 trial,^7^ thereby reinforcing the clinical value of this therapeutic approach even in less selected populations.

Compared to registrational and real-world studies, the MALVA cohort represents a clinically heterogeneous population, with features that place it between highly selected trial cohorts and broader real-world samples. For instance, the French AVENANCE study included a more fragile population (median age 73, predominance of carboplatin use),^8^ while the Italian READY^9^ study presented an intermediate profile with a good distribution of platinum-based regimens and a majority ECOG 0 status. These differences may partially account for the variability in clinical outcomes observed across studies, such as the higher median OS in READY (26.2 months) and the relatively lower survival in AVENANCE (Supplemental Table S6).

In our study, the overall response rate (ORR) was 26.75% and the disease control rate (DCR) was 69.30%, indicating that a substantial proportion of patients achieved clinical benefit. Responders (CR+PR) showed significantly longer OS (not reached) compared to patients with disease progression (OS 7.2 months), confirming the prognostic relevance of response to therapy (Supplemental Table S6).

Univariate and multivariate analyses identified ECOG ≥1, bone metastases, brain metastases, and concomitant steroid use as independent predictors of poor prognosis. These findings are in line with existing literature, highlighting the detrimental impact of PS decline, aggressive metastatic patterns, and immunosuppressive therapies on immunotherapy efficacy.^10^

A major strength of the study lies in the subgroup analysis, which defined a simple exploratory prognostic stratification score based on three variables—ECOG ≥1, presence of bone metastases, and steroid use prior to Avelumab. This stratified patients into distinct risk groups with markedly different outcomes: patients with no adverse factors (score 0) had a median OS of 39.9 months and an ORR of 33.6%, whereas those with two or more negative factors (score 2-3) had a median OS of 8.4 months and an ORR of only 6.9%. Importantly, these differences were not attributable to imbalances in other variables such as age, sex, tumor site, histology, or prior treatments, suggesting that the exploratory prognostic stratification score reflects true prognostic differences rather than selection bias (Supplemental Table S4).

The predictive role of ECOG performance status is well-established in oncology and particularly relevant in the context of immunotherapy.^11^ A higher ECOG score reflects impaired functional capacity, often correlating with comorbid conditions, frailty, and reduced tolerance to systemic treatments.^12^ In clinical trials and real-world studies alike, ECOG ≥1 has consistently been associated with poorer survival outcomes and lower treatment efficacy.^13^ This may be due to multiple factors, including reduced immune competence, altered pharmacokinetics, and the inability to complete or respond optimally to immunotherapeutic regimens. Our findings reaffirm the prognostic impact of ECOG status, as patients with ECOG ≥1 in our cohort experienced significantly shorter overall survival compared to those with ECOG 0.

Similarly, the presence of bone metastases has long been recognized as a marker of aggressive disease biology and poor prognosis across various tumor types, including urothelial carcinoma.^14^ Bone metastases often create an immunosuppressive niche through the secretion of transforming growth factor-beta (TGF-β), interleukins, and other soluble factors by the tumor and the surrounding bone microenvironment.^15^ This altered milieu can impair antigen presentation, T-cell infiltration, and overall immune activation—mechanisms that are crucial for the efficacy of immune checkpoint inhibitors.^16^ Moreover, bone involvement frequently leads to skeletal-related events (SREs), such as pathological fractures, spinal cord compression, and severe pain, all of which further compromise patient performance status and limit therapeutic options.^17^

Pre-treatment use of systemic corticosteroids represents another negative prognostic factor identified in our study.^18^ While steroids are often necessary for the management of cancer- or treatment-related symptoms, their immunosuppressive properties can counteract the mechanism of action of immune checkpoint blockade. Steroids reduce T-cell proliferation, diminish cytokine production, and can impair the activation and expansion of tumor-specific immune responses. These effects are particularly detrimental when steroids are administered before or at the onset of immunotherapy.^19^ Even low doses of corticosteroids—particularly when used for non-oncologic indications such as autoimmune diseases, chronic obstructive pulmonary disease (COPD), or brain edema—have been associated with inferior outcomes in patients treated with PD-1/PD-L1 inhibitors.^20^

It is also important to consider that the need for corticosteroids—especially at the initiation of Avelumab—may itself be a marker of poor prognosis. In many cases, steroids are prescribed to manage cancer-related symptoms such as fatigue, anorexia, brain edema, or pain, which often reflect a higher tumor burden or more advanced disease.^21^ Therefore, beyond their direct immunosuppressive effects, early steroid use may identify a subgroup of patients with more aggressive clinical presentations and limited physiological reserve. This dual role—as both a therapeutic agent and a surrogate marker of disease severity—could partially explain the observed association between baseline steroid use and inferior survival outcomes in our cohort.

Despite being partly retrospective, the study’s multicenter design and large sample size enhance its external validity. Limitations include potential selection bias, lack of molecular biomarker data (eg, PD-L1, TMB), incomplete information on steroid dosing, the absence of information on the timing of avelumab infusions, PFS that in our study was investigator-assessed, included cases of clinical progression without standardized criteria, and lacked central radiology review, which limits the objectivity of this endpoint and the lack of centralized pathology review. In addition, Histological diagnoses were based on local assessments, and cases described as “urothelial carcinoma with histologic subtypes” may have been subject to interobserver variability and a very small number of patients with ECOG ≥2, which restricts the ability to draw firm conclusions in this clinically fragile subgroup. Nonetheless, the relatively low incidence of severe immune-related adverse events and the manageable toxicity profile observed align with the safety data reported in pivotal trials. Finally, In our study, we explored the prognostic role of baseline clinical factors and proposed a simple, pragmatic stratification of patients according to ECOG performance status, presence of bone metastases, and corticosteroid use. We acknowledge that this approach does not follow the methodological standards for the development of validated prognostic models (eg, TRIPOD) and should therefore be interpreted as an exploratory, post-hoc analysis rather than a definitive “prognostic score.” Each variable was considered as present or absent without weighting, and the resulting groups should be regarded as descriptive rather than predictive.

Brain metastases, although strongly associated with worse prognosis (HR 7.02), were not included in the stratification because of the very limited number of affected patients in our cohort, which would have led to unstable subgroup estimates. We have now clarified this rationale. The absence of liver metastases as an independent prognostic factor in our model contrasts with prior evidence; this discrepancy may reflect differences in population characteristics, treatment setting, or limited statistical power, and deserves further investigation.

Finally, we recognize that other clinically relevant variables such as baseline anemia or systemic inflammatory indexes (eg, NLR, PNR, SII) were not systematically available across centers and could not be incorporated into our analysis. This represents an important limitation, and future prospective studies integrating both clinical and biological markers will be necessary to refine prognostic stratification in this setting.

In summary, our findings confirm the real-world efficacy of Avelumab maintenance in urothelial cancer and underscore the importance of patient stratification. The proposed exploratory prognostic stratification score may assist clinicians in identifying patients more likely to benefit from maintenance immunotherapy and in tailoring alternative strategies for those at higher risk. Prospective validation and integration with biological markers are warranted to further personalize treatment in this setting.

## Supplementary Material

oyaf388_Supplementary_Data

## Data Availability

The datasets generated and analyzed during the current study are not publicly available due to privacy regulations but are available from the corresponding author on reasonable request.
