# Supplementary material for: Real-world effectiveness of Avelumab maintenance in advanced urothelial carcinoma: results from the Italian multicenter MALVA study (Meet-URO 25)
Source: Oncologist. 2025 Nov 20;30(12):oyaf388. doi: 10.1093/oncolo/oyaf388 (PMC12680435; doi:10.1093/oncolo/oyaf388)

**Supplemental Figure S1. Forest Plot of Clinical and Biological Variables**


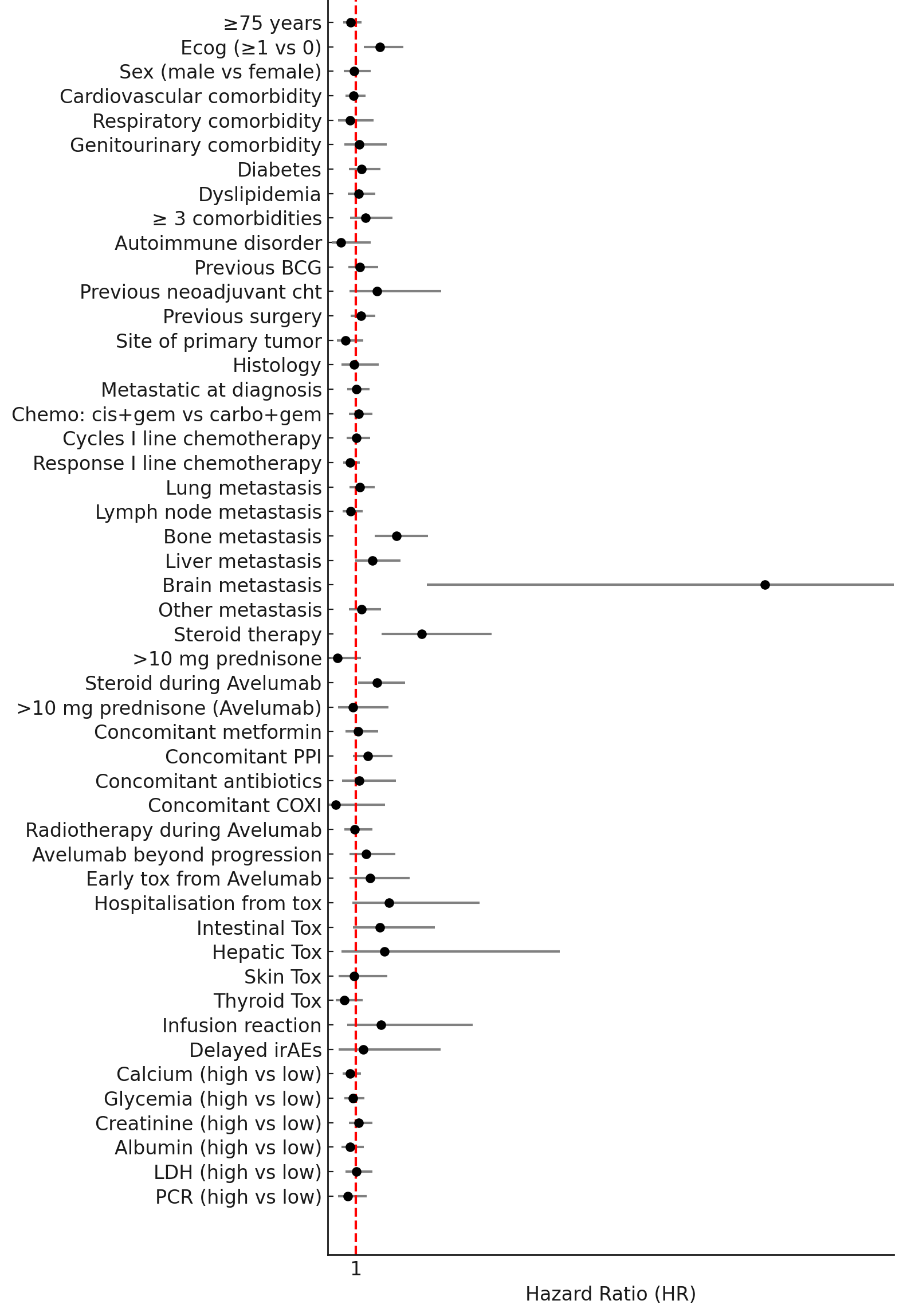


**Supplemental Figure S2. clinical responses by score groups**


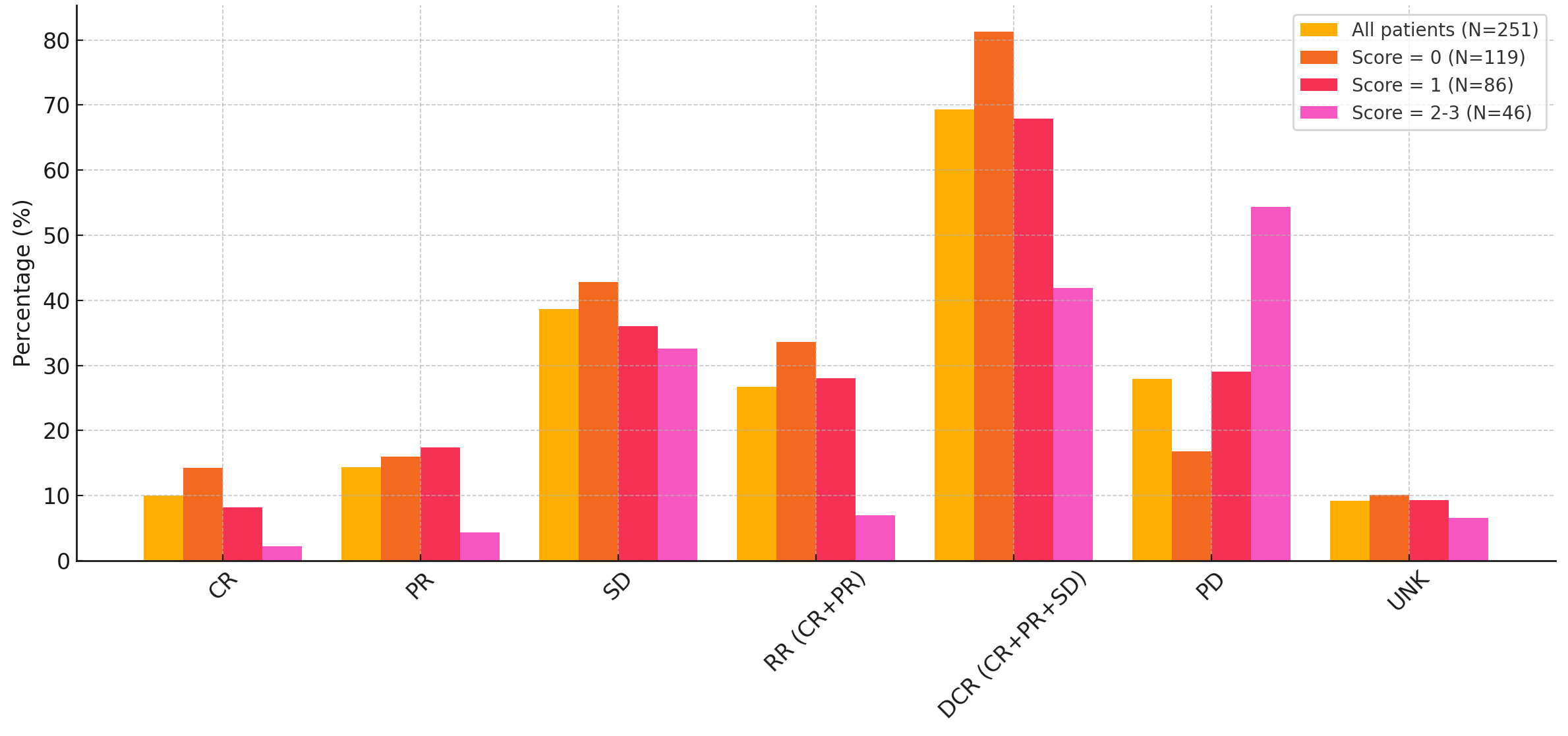


**Supplemental Figure S3. Progression free survival by score group**


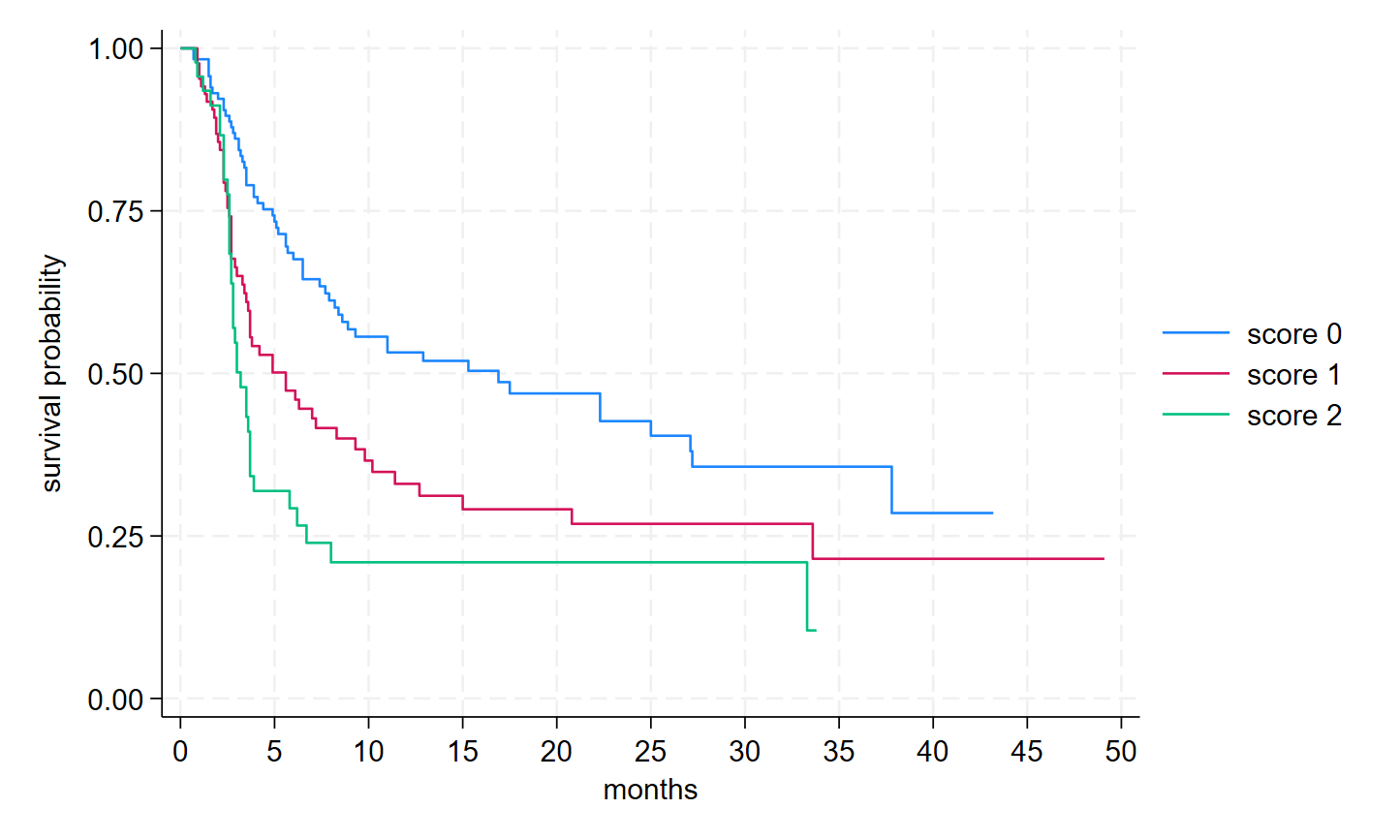

Supplement: oyaf388_Supplementary_Data [file oyaf388_supplementary_data.zip › Supplementary Figures.docx]
