# Supplementary material for: Real-world effectiveness of Avelumab maintenance in advanced urothelial carcinoma: results from the Italian multicenter MALVA study (Meet-URO 25)
Source: Oncologist. 2025 Nov 20;30(12):oyaf388. doi: 10.1093/oncolo/oyaf388 (PMC12680435; doi:10.1093/oncolo/oyaf388)
Supplement: oyaf388_Supplementary_Data [file oyaf388_supplementary_data.zip › Supplementary Tables.docx]

**Supplemental Table S1: Best response, PFS and OS during avelumab therapy.**

|  | **All patients**  **(N=251)** |
| --- | --- |
| RC | 25 (9.96) |
| PR | 36 (14.34) |
| SD | 97 (38.65) |
| RR (RC+PR) | 61 (26.75) |
| DCR (RC+PR + SD) | 158 (69.30) |
| PD | 70 (27.89) |
| UNK | 23 (9.16) |
| PFS  M-months (95% IC) | 7  (5.6-9.3) |
| OS  M-months (95% IC) | 22.4  (16.5-36.6) |
| OS  M-months (95% IC) responders (RC+PR)  M-months (95% IC) non-responders (SD+PD) | NR (35.5-NR)  14.1 (11.5-18.5) |
| OS  M-months (95% IC) responders (RC+PR+SD)  M-months (95% IC) non-responders (PD) | 38.6 (27.6-NR)  7.2 (5-11.9) |

**Supplemental Table S2. Treatment discontinuation and subsequent therapy**

| **Variables** | **Total 251 patients** |
| --- | --- |
| Avelumab maintenance therapy ongoing, n (%) | 161 (35.8) |
| Avelumab maintenance therapy discontinued, n (%) | 161 (58.96) |
| Reason for discontinuation, n (%)  Disease progression  Toxicity*  Unknown  Other  *Including three deaths probably related to avelumab | 130 (80.75)  26 (16,15)  4 (2.48)  1 (0.62) |
| Subsequent therapy  Enfortumab Vedotin  Paclitaxel  Carboplatin  Vinflunine  Sacituzumab Govitecan  Paclitaxel+ carboplatin  Gemcitabine + cisplatin  Docetaxel  Gemcitabine+carboplatin  Erdafitinib | 94 (37.05)  59 (62.77)  10 (10.64)  7 (7.45) 7 (7.45)  3 (3.19)  2 (2.13)  2 (2.13)  2 (2.13)  1 (1.06)  1 (1.06) |

**Supplemental Table S3. Steroid use and avelumab**

|  | **Number** | **Dosage > 10 mg prednisone** |
| --- | --- | --- |
| **Steroid use before to start avelumab**  Type of steroid  Prednisone  Desametasone  Metilprednisone  Betametasone  **Motivation for steroid use before avelumab**  brain metastases  bone pain  fatigue  anorexia  COPD | 19 (7.57)  11  6  1  1  3  10  2  1  1 | 13 (68.42)  3  8  1  0  0 |
| **Steroid use during avelumab**  Type of steroid  Prednisone  Desametasone  Metilprednisone  Betametasone | 34 (13.55)  28  3  3  0 | 21 (61.76) |

**Supplemental Table S4. Patient baseline characteristics according to subgroup patients.**

| **Variables** | **Total**  **251 patients** | **Subgroup patients** | | |
| --- | --- | --- | --- | --- |
|  |  | **Score = 0**  **(N = 119)** | **Score = 1**  **(N = 86)** | **Score = 2-3**  **(N = 46)** |
| Age, median (range), years  ≥75 years; n (%) | 72 (38-88)  96 (38.2) | 71 (38-84)  96 (38.2) | 75 (48-86)  96 (38.2) | 71 (43-88)  96 (38.2) |
| Sex, n (%)  Male  Female | 206 (82.07)  45 (17.93) | 94 (78.99)  25 (21.01) | 76 (88.37)  10 (11.63) | 36 (78.26)  10 (21.74) |
| ECOG performance status, n (%)  0  1  ≥2 | 152 (60.56)  89 (35.46)  10 (3.98) | 119 (100.0)  0  0 | 28 (35.56)  55 (63.94)  3 (3.49) | 5 (10.87)  34 (73.91)  7 () |
| Primary tumor site, n (%)  Bladder  Upper urinary tract | 186 (74.10)  65 (25.90) |  |  |  |
| Hystology  Urothelial  urothelial carcinoma with histologic subtypes | 230 (91.63)  21 (8.37) | 106 (89.08)  13 (10.92) | 80 (93.02)  6 (6.98) | 44 (95.65)  2 (4.35) |
| Site of metastasis, n (%)  Lung  Bone  Liver  Lymph node  Brain  Other | 78 (31.08)  66 (26.29)  35 (13.44)  192 (76.49)  3 (1.23)  45 (18.4) | 46 (38.66)  0  8 (6.72)  92 (77.31)  0  19 (15.97) | 17 (19.77)  22 (25.58)  18 (20.93)  66 (76.74)  1 (1.16)  22 (25.58) | 15 (32.61)  44 (95.65)  9 (19.57)  34 (73.91)  2 (4.35)  4 (8.70) |
| Metastatic at diagnosis n (%)  Yes  No | 108 (43.03)  143 (56.97) | 55 (46.22)  64 (53.78) | 29 (33.72)  57 (66.28) | 24 (52.17)  22 (47.83) |
| Comorbidities, n (%)  Cardiovascular  Respiratory  Genitourinary  Diabetes  Dyslipidaemia  Autoimmune disease  ≥ 3 comorbidities | 164 (65.34)  21 (8.37)  23 (9.16)  44 (17.53)  64 (25.50)  14 (5.58)  23 (9.16) | 75 (63.03)  9 (7.56)  11 (9.24)  19 (15.97)  28 (23.53)  9 (7.56)  11 (9.24) | 60 (69.77)  8 (9,30=  7 (8.14)  19 (22.09)  24 (27.91)  3 (3.49)  12 (13.95) | 29 (63.04)  4 (8.70)  5 (10.87)  6 (13.04)  12 (26.09)  2 (4.35)  7 (15.22) |
| Prior treatment, n (%)  Surgery  Radio chemotherapy | 109 (43.43)  2 () | 53 (44.54)  1 () | 40 (46.51)  0 | 16 (34.78)  1 () |
| Prior chemotherapy regimen, n (%)  Gemcitabine + cisplatin  Gemcitabine + carboplatin | 117 (46.80)  133 (53.20) | 52 (43.7)  67 (56.30) | 55 (63.95)  31 (36.05) | 26 (57.68)  19 (42.22) |
| Number of cycles of prior chemotherapy, n (%)  4  5  6 | 179 (71.73)  23 (9.16)  49 (19.52) | 91 (76.47)  9 (7.56)  19 (15.97) | 53 (61.63)  9 (10.47)  24 (27.91) | 35 (76.09)  5 (10.87)  6 (13.04) |
| Best overall response to prior chemotherapy, n (%)  CR  PR  SD | 12 (4.78)  134 (53.39)  105 (41.83) | 8 (6.72)  62 (52.10)  49 (41.18) | 3 (3.49)  49 (56.98)  34 (39.53) | 1 (2.17)  23 (50.00)  22 (47.83) |

**Supplemental Table S5: Best response, PFS and OS according to subgroup patients.**

|  | **All patients**  **(N=251)** | **Subgroup patients** | | |
| --- | --- | --- | --- | --- |
|  |  | **Score = 0**  **(N = 119)** | **Score = 1**  **(N = 86)** | **Score = 2-3**  **(N = 46)** |
| CR | 25 (9.96) | 17 (14.29) | 7 (8.14) | 1 (2.17) |
| PR | 36 (14.34) | 19 (15.97) | 15 (17.44) | 2 (4.35) |
| SD | 97 (38.65) | 51 (42.86) | 31 (36.05) | 15 (32.61) |
| RR (RC+PR) | 61 (26.75) | 36 (33.64) | 22 (28.01) | 3 (6.98) |
| DCR (RC+PR + SD) | 158 (69.30) | 87 (81.31) | 53 (67.95) | 18 (41.86) |
| PD | 70 (27.89) | 20 (16.81) | 25 (29.07) | 25 (54.35) |
| UNK | 23 (9.16) | 12 (10.08) | 8 (9.30) | 3 (6.52) |
| PFS  M-months (95% IC) | 7  (5.6-9.3) | 16.9  (8.4-27.1) | 5.6  (3.5-9.3) | 3.2  (2.7-3.7) |
| OS  M-months (95% IC) | 22.4  (16.5-36.6) | 39.9  (36.6-NR) | 14.1  (10.3-19.7) | 8.4  (5.6-11.9) |

**Supplemental Table S6. Summary of relevant experience with avelumab**

| **Study** | **Number of Patients** | **Median**  **Overall Survival** | **Median**  **Progression-Free Survival** | **Tumor Response** |
| --- | --- | --- | --- | --- |
| **JAVELIN Bladder 100** | 350 | 21.4 months  (95% CI: 18.9–26.1) | 3.7 months (95% CI: 2.1–4.2) | CR: 6%; PR: 4%; SD: 13% |
| **AVENANCE (France)** | 593 | 20.7 months  (95% CI: 15.2–NE) | 5.7 months (95% CI: 5.1–7.9) | CR: 3%; PR: 12%; SD: 38% |
| **READY (Italy)** | 414 | 26.2 months  (95% CI: 19.97–NE) | 8.1 months (95% CI: 5.6–8.9) | Not specified |
| **MALVA Study (Meet-URO 25) (Italy)** | 251 | 22.4 months  (95% CI: 16.5–36.6) | 7.0 months (95% CI: 5.6–9.3) | CR: 10%; PR: 14%; SD: 39% |
| - **CR**: Complete Response - **PR**: Partial Response - **SD**: Stable Disease - **NE**: Not Estimable (due to immature data or ongoing follow-up) | | | | |
